# A real‐world study of adjuvant anti‐PD ‐1 immunotherapy on stage III melanoma with BRAF, NRAS, and KIT mutations

**DOI:** 10.1002/cam4.6234

**Published:** 2023-07-05

**Authors:** Wei Sun, Yu Xu, WangJun Yan, ChunMeng Wang, Tu Hu, ZhiGuo Luo, XiaoWei Zhang, Xin Liu, Yong Chen

**Affiliations:** ^1^ Department of Musculoskeletal Surgery, Fudan University Shanghai Cancer Center; Department of Oncology, Shanghai Medical College Fudan University Shanghai China; ^2^ Department of gastrointestinal medical oncology, Fudan University Shanghai Cancer Center; Department of Oncology, Shanghai Medical College Fudan University Shanghai China; ^3^ Department of Head&Neck tumors and Neuroendocrine tumors, Fudan University Shanghai Cancer Center; Department of Oncology, Shanghai Medical College Fudan University Shanghai China

## Abstract

**Background:**

Melanoma frequently harbors BRAF, NRAS, or KIT mutations which influence both tumor development and treatment strategies. For example, it is still controversial whether adjuvant anti‐PD‐1 monotherapy or BRAF/MEK inhibitors may better improve the survival for resected BRAF‐mutant melanoma. Furthermore, outcomes for melanoma with NRAS and KIT mutation receiving adjuvant immunotherapy remain unclear.

**Methods:**

One hundred seventy‐four stage III melanoma patients who underwent radical surgery in Fudan University Shanghai Cancer Center (FUSCC) during January 2017 to December 2021 were included in this real‐world study. Patients were followed up until death or May 30th, 2022. Pearson's chi‐squared test or Fisher's exact test was performed for univariable analysis of the different category groups. Log‐rank analysis was used to identify the prognostic factors for disease‐free survival (DFS).

**Results:**

There were 41 (23.6%) patients with BRAF mutation, 31 (17.8%) with NRAS mutation, 17 (9.8%) with KIT mutation, and 85 (48.9%) wild‐type patients without either genomic alteration of those three genes. Most ( *n*  = 118, 67.8%) of them were acral melanoma, while 45 (25.9%) were cutaneous subtype, and 11 were (6.3%) primary unknown. Among them, 115 (66.1%) patients received pembrolizumab or toripalimab monotherapy as adjuvant therapy; 22 (12.6%) patients received high‐dose interferon (IFN), and 37 (21.3%) patients were just for observation. There was no statistical difference in clinicopathologic factors between anti‐PD‐1 group and IFN/OBS group. Of all the enrolled patients, anti‐PD‐1 group had a better DFS than IFN/OBS group ( *p*  = 0.039). In anti‐PD‐1 group, patients with BRAF or NRAS mutations had poorer DFS than wild‐type group. No survival difference was found among patients harboring different gene mutations in IFN/OBS group. In wild‐type patients, anti‐PD‐1 group had a better DFS than IFN/OBS group ( *p*  = 0.003), while no survival benefits were found for patients with BRAF, NRAS, or KIT mutations.

**Conclusion:**

Although anti‐PD‐1 adjuvant therapy provides a better DFS in the general population and in wild‐type patients, patients with BRAF, KIT or, especially, NRAS mutation may not benefit further from immunotherapy than conventional IFN treatment or observation.

## 1 INTRODUCTION

Melanoma is an aggressive and genetically heterogenous disease. It frequently harbors BRAF, NRAS, or KIT mutations which influence both tumor development and treatment strategies. Nearly half of cutaneous melanoma and 5%–10% of other subtypes possess a BRAF^V600^ mutation.^1^, ^2^, ^3^ In the adjuvant setting, BRAF and MEK inhibitors combination has been approved for BRAF^V600^‐mut patients with Stage III disease, while anti‐PD1 monotherapy approved for both mutant and wild‐type melanoma. The KEYNOT‐054 phase III trial assessed pembrolizumab versus placebo in patients with resected high‐risk stage III melanoma. In its recurrence‐free survival (RFS) and distant metastasis‐free survival (DMFS) subgroup analysis, both BRAF^V600^ mutant and wild‐type patients favored pembrolizumab as adjuvant therapy.^4^, ^5^ The CheckMate 238 trial, which evaluated the efficacy of nivolumab versus ipilimumab for adjuvant therapy, also showed benefit in survival regardless of BRAF status.^6^ Similar results were concluded for resected Stage IV melanoma in the IMMUNED phase II trial.^6^ On the contrary, combination therapies with the BRAF/MEK inhibitors (dabrafenib/trametinib) have shown a longer duration of survival than placebo in stage III melanoma (5‐year disease‐free survival (DFS), 52% versus 36%; hazard ratio (HR), 0.51).^7^ In the absence of comparative studies, it is still controversial whether adjuvant immunotherapy (IO) with PD‐1 checkpoint blockade, or targeted therapy with BRAF/MEK inhibitors, may better improve the survival of BRAF‐mutant patients.

NRAS‐mutant melanoma is a distinct cohort which comprises 13% to 20% of this disease, and appears to confer a poor prognosis.^8^, ^9^ In contrast with BRAF mutation, no effective small molecule inhibitor specifically targeting NRAS has been approved. Although no significant difference have reached both in objective response rates (ORR) and overall survival (OS), previous studies of IL‐2 and ipilimumab do have reported numerical improvement in NRAS‐mutated advanced melanoma patients.^10^, ^11^ A multicenter retrospective study suggested particularly a remarkable benefit from anti‐PD‐1/PD‐L1 therapy in advanced melanoma patients with NRAS mutations compared with other genetic alternation.^12^ By contrast, another retrospective study for checkpoint inhibitor showed a worse survival for NRAS‐mutant melanoma than NRAS wildtype (21 vs. 33 months, *p* = 0.034), although both groups showed comparable response rates.^13^ Asian melanoma patients with high incidence of acral and mucosal subtype were reported with lower efficacy for immunotherapy, due to low tumor mutation burden and PD‐L1 expression, but high proportion of copy number variations and chromosomal structure variations. In a pooled analysis study of four Asian clinical trials on anti‐PD‐1 monotherapy, advanced melanoma with NRAS mutations had lower response rate and poorer prognosis, especially in acral and mucosal subtypes.^14^

KIT alterations are relatively rare in Caucasian populations but more frequent in Asian patients. Preclinical studies have demonstrated that hot spot mutations, mostly substitutions in exons 11 and 13, result in sustained activation of KIT and its downstream signal cascades, including the MEK/ERK, PI3K/AKT, and JAK/STAT pathways.^15^, ^16^ Several studies and case reports have revealed the therapeutic value of c‐kit inhibitors, such as imatinib, nilotinib, and dasatinib, mono or combing with anti‐PD‐1 antibodies.^17^, ^18^, ^19^ However, the influence of KIT mutations on the efficacy of anti‐PD‐1 monotherapy in melanoma has not been extensively explored.

This real‐world study was conducted to assess the association of BRAF, NRAS, or KIT mutations with outcome in melanoma receiving adjuvant anti‐PD‐1 monotherapy. Wild‐type patients and the patients received high‐dose interferon (HD‐IFN) or observation were set as control in the analysis.

## 2 PATIENTS AND METHODS

Eligible consecutive patients with pathologically confirmed stage III melanoma who underwent radical surgery in Fudan University Shanghai Cancer Center (FUSCC) from January 2017 to December 2021 were included in the current real‐world study. All patients with positive sentinel lymph node underwent completion lymph node dissection within 1 month after biopsy. Pathologic nodal (pN) stage and pathological stage were defined according to the 8th edition of the American Joint Committee on Cancer (AJCC) cancer staging manual.^20^ The pathological methods to detect the primary tumors and lymph node metastases were similar to those used in our prior study.^21^, ^22^ Cases with baseline distant metastasis and mucosal melanoma were excluded.

NRAS, BRAF, and KIT mutations were detected by PCR from formalin‐fixed paraffin‐embedded (FFPE) tissue. Clinicopathologic data such as subtype, T stage, ulceration, nodal involvement, and adjuvant therapy were collected for survival analyses. In anti‐PD‐1 immunotherapy (IO) group, patients received pembrolizumab or toripalimab, with recommended dosage, respectively. In HD‐IFN group, patients received IFN α‐2b 15 MU/m^2^, iv, days 1–5, weeks 1–4. Patients received adjuvant therapy at most 1 year or until recurrence. Patients were monitored through clinical examination such as routine physical checkups, ultrasound, CT and/or MRI every 3 months for the first 2 years, every 6 months for 3–5 years, and then annually. Recurrence or metastasis was confirmed by pathology or imaging follow‐up. Patients were followed up until death or May 30th, 2022. DFS was defined as the time interval from radical surgery to local recurrence or distant metastasis.

Pearson's chi‐squared test or Fisher's exact test was performed for univariable analysis of the different category groups. Kaplan–Meier estimation and Log‐rank analysis were used to identify the prognostic factors for DFS. A *p* value of 0.05 or less (2‐sided test) was considered significant. All analyses were conducted with R software, version 4.2.0 (http://www.R‐project.org) and SPSS (version 22.0; SPSS Company, Chicago, IL) software.

Each participant signed an informed consent document during the preoperative conversation. This study was approved by the Medical Ethics Committee of FUSCC, and all methods were performed in accordance with the Declaration of Helsinki and the relevant guidelines and regulations.

## 3 RESULTS

### 3.1 Baseline characteristics

A total of 174 melanoma patients were included in the current study, including 118 (67.8%) acral subtype, 45 (25.9) cutaneous subtype, and 11 (6.3%) primary unknown melanoma. Seventy‐six (43.68%) patients were female, and 98 patients (56.32%) were male. The genomic alternation included 41 (23.6%) BRAF mutation, 31 (17.8%) NRAS mutation, 17 (9.8%) KIT mutation, and 85 (48.9%) wild type of those three genes. All BRAF alternation were V600E mutations on exon 15. Among NRAS‐mutant patients, 9 (29.0%) were G12 mutation which occurred on exon 2, 21 (67.1%) Q61 mutations on exon 3, while one (3.2%) with NRAS amplification. KIT mutations were diverse, occurring on exon 11 (11, 64.8%), exon 13 (3, 17.6%), and exon 17 (3, 17.6%). The most common (8, 47.1%) KIT mutation type was L567P on exon 11. (Table S1) No patient has two or three mutations at the same time. As shown in Table 1, no statistical difference was found between each genotype group in gender, T stage, ulceration, nodal involvement, N stage, stage III subgroup, or adjuvant therapy. Meanwhile, patients with BRAF mutation were younger than wild‐type patients (*p* = 0.013). BRAF mutations were more common (23, 56.1%) in cutaneous subtype, while most NRAS (29, 93.5%) and KIT (16, 94.1%) mutations were found in acral subtype.

**TABLE 1:** Relationships between tumor mutations and clinicopathologic features.

	WT (%)	BRAF (%)		NRAS (%)		KIT (%)	
	*n* = 85	*n* = 41	*p*	*n* = 31	*p*	*n* = 17	*p*
Gender			0.186		0.436		0.280
Female	37 (43.5%)	23 (56.1%)		11 (35.5%)		5 (29.4%)	
Male	48 (56.5%)	18 (43.9%)		20 (64.5%)		12 (70.6%)	
Age (median)			0.013		0.374		0.244
< 60 yrs.	38 (44.7%)	28 (68.3%)		11 (35.5%)		5 (29.4%)	
> = 60 yrs	47 (55.3%)	13 (31.7%)		20 (64.5%)		12 (70.6%)	
Subtype			0.002		**0.018**		0.063
Acral	58 (68.2%)	15 (36.6%)		29 (93.5%)		16 (94.1%)	
Cutaneous	21 (24.7%)	23 (56.1%)		1 (3.2%)		0 (0%)	
Unknown primary	6 (7.1%)	3 (7.3%)		1 (3.2%)		1 (5.9%)	
T stage			0.207		0.099		0.993
T0	6 (7.1%)	3 (7.3%)		1 (3.2%)		1 (5.9%)	
T1	5 (5.9%)	0 (0%)		0 (0%)		1 (5.9%)	
T2	7 (8.2%)	8 (19.5%)		6 (19.4%)		2 (11.8%)	
T3	27 (31.8%)	10 (24.4%)		5 (16.1%)		5 (29.4%)	
T4	40 (47.1%)	20 (48.8%)		19 (61.3%)		8 (47.1%)	
Ulceration			0.885		0.186		0.535
No	26 (32.9%)	12 (31.6%)		6 (20.0%)		4 (25.0%)	
Yes	53 (67.1%)	26 (68.4%)		24 (80.0%)		12 (75.0%)	
Nodal involvement			0.631		0.165		0.125
Micrometastasis	48 (56.5%)	25 (61.0%)		13 (41.9%)		13 (76.5%)	
Macrometastasis	37 (43.5%)	16 (39.0%)		18 (58.1%)		4 (23.5%)	
N stage			0.136		0.087		0.091
N1	45 (52.9%)	14 (34.1%)		10 (32.3%)		9 (52.9%)	
N2	24 (28.2%)	17 (41.5%)		10 (32.3%)		8 (47.1%)	
N3	16 (18.8%)	10 (24.4%)		11 (35.5%)		0 (0%)	
Stage III subgroup			0.927		0.129		0.536
IIIA	9 (10.6%)	3 (7.3%)		2 (6.5%)		2 (11.8%)	
IIIB	14 (16.5%)	8 (19.5%)		3 (9.7%)		2 (11.8%)	
IIIC	54 (63.5%)	26 (63.4%)		18 (58.1%)		13 (76.5%)	
IIID	8 (9.4%)	4 (9.8%)		8 (25.8%)		0 (0%)	
Adjuvant therapy			0.142		0.863		0.923
Anti‐PD1	59 (69.4%)	23 (56.1%)		21 (67.7%)		12 (70.6%)	
IFN or OBS	26 (30.6%)	18 (43.9%)		10 (32.3%)		5 (29.4%)	
Relapse Mode (initial)			**0.030**		**0.002**		0.746
Regional	15 (17.6%)	10 (24.4%)		15 (48.4%)		3 (17.6%)	
Systemic	18 (21.2%)	16 (39.0%)		7 (22.6%)		5 (29.4%)	
No relapse	52 (61.2%)	15 (36.6%)		9 (29.0%)		9 (52.9%)	

*p* value < 0.05 are indicated in bold.

### 3.2 Survival analysis in different treatment groups

**FIGURE 1:**
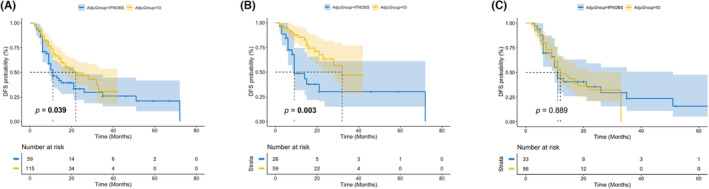
Kaplan–Meier plot curves for the disease‐free survival (DFS) of patients in anti‐PD‐1 immunotherapy group and IFN/OBS group. (A) All patients, (B) wild‐type patients, (C) mutated patients.

Among all the cases, 115 (66.1%) patients received anti‐PD‐1 antibody monotherapy as adjuvant therapy, 22 (12.6%) patients received HD‐IFN, and 37 (21.3%) patients were just for observation. As HD‐IFN is no longer used as an adjuvant therapy owing to the marginal benefit in survival, we combined the IFN group and observation group into one group (IFN/OBS) for the following analysis.^23^, ^24^, ^25^ There was no statistical difference in clinicopathologic data, including gender, age, subtype, gene mutation, ulceration, and tumor stage, between IO group and IFN/OBS group. The median follow‐up time was 21 months in IO group and 46 months in IFN/OBS group. Reginal relapse occurred in 26 (22.6%) patients and distant metastasis was found in 25 (21.7%) patients in the IO group during the follow‐up, while the corresponding number in IFN/OBS group was 17 (28.8%) and 21 (35.6%), respectively (*p* = 0.036; Table 2). Therefore, IO group had a better DFS than IFN/OBS group (median DFS: 22 vs. 11 months; *p* = 0.039; Figure 1A). In wild‐type group, the IO group also remarked a better DFS than the IFN/OBS group (median DFS: 32 vs. 9 months; *p* = 0.003, Figure 1B). However, in mutated group, no statistical survival benefit was found in the IO group than in the IFN/OBS group (median DFS: 11 vs. 10 months; *p* = 0.889; Figure 1C).

**TABLE 2:** Relationships between different treatment groups and clinicopathologic features.

	PD‐1 (%) *n* = 115	IFN or OBS (%) *n* = 59	*p*
Gender			0.124
Female	55 (47.8%)	21 (35.6%)	
Male	60 (52.2%)	38 (64.4%)	
Age (median)			0.368
<60 yrs.	57 (49.6%)	25 (42.4%)	
> = 60 yrs	58 (50.4%)	34 (57.6%)	
Subtype			0.492
Acral	75 (65.2%)	43 (72.9%)	
Cutaneous	33 (28.7%)	12 (20.3%)	
Unknown primary	7 (6.1%)	4 (6.8%)	
Gene mutation			0.487
BRAF	23 (10.0%)	18 (30.5%)	
NRAS	21 (18.3%)	10 (16.9%)	
KIT	12 (10.4%)	5 (8.5%)	
Wild type	59 (51.3%)	26 (44.1%)	
T stage			0.917
T0	7 (6.1%)	4 (6.8%)	
T1	5 (4.3%)	1 (1.7%)	
T2	15 (13.0%)	8 (13.6%)	
T3	30 (26.1%)	17 (28.8%)	
T4	58 (50.4%)	29 (49.2%)	
Ulceration			0.059
No	71 (65.7%)	44 (80.0%)	
Yes	37 (34.3%)	11 (20.0%)	
Nodal involvement			0.612
Micrometastasis	67 (58.3%)	32 (54.2%)	
Macrometastasis	48 (41.7%)	27 (45.8%)	
N stage			0.093
N1	49 (42.6%)	29 (49.2%)	
N2	36 (31.3%)	23 (39.0%)	
N3	30 (26.1%)	7 (11.9%)	
Stage III subgroup			0.192
IIIA	12 (10.4%)	4 (6.8%)	
IIIB	14 (12.2%)	13 (22.0%)	
IIIC	73 (63.5%)	38 (64.4%)	
IIID	16 (13.9%)	4 (6.8%)	
Relapse Mode (initial)			**0.036**
Regional	26 (22.6%)	17 (28.8%)	
Systemic	25 (21.7%)	21 (35.6%)	
No relapse	64 (55.7%)	21 (35.6%)	

*p* value < 0.05 are indicated in bold.

### 3.3 Survival analysis in different gene mutation subgroups

**FIGURE 2:**
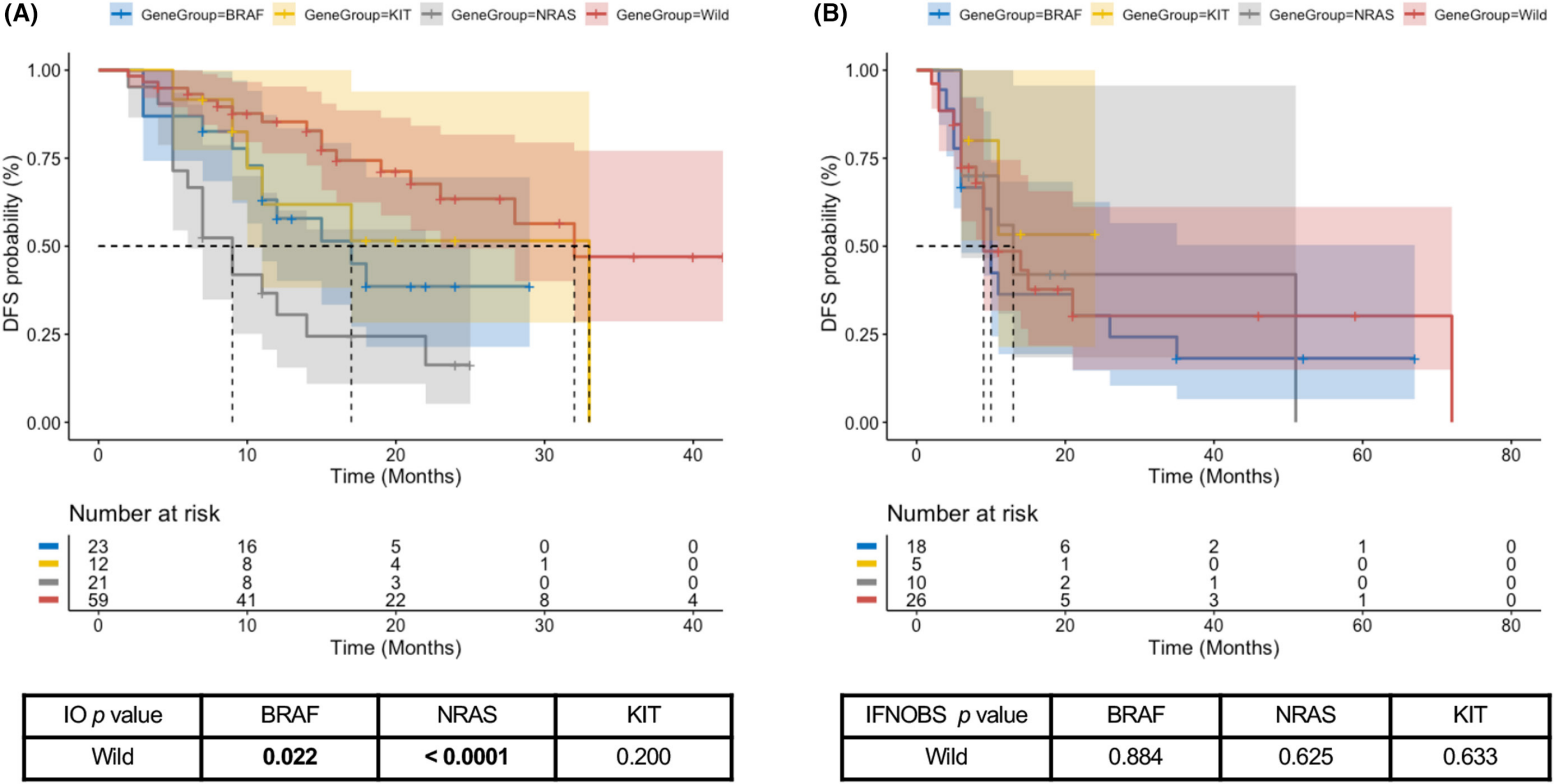
Kaplan–Meier plot curves for the disease‐free survival (DFS) of patients harbor different gene mutations who received anti‐PD‐1 immunotherapy (A) or IFN/OBS (B).

Comparing with the disease‐free survival rate of 61.2% (*n* = 52) in wild‐type group, only 36.6% (*n* = 15) BRAF‐mutant patients (*p* = 0.030) and 29.0% (*n* = 9) NRAS‐mutant patients (*p* = 0.002) had no recurrence during the follow‐up. BRAF‐mutant patients noted the highest systemic metastasis rate of 39% (*n* = 16), while nearly half (48.4%, *n* = 15) of NRAS‐mutant patients suffered regional relapse. Among patients receiving anti‐PD‐1 monotherapy, wild‐type patients showed the best survival with a median DFS of 32 months. The median DFS of those with BRAF and NRAS mutations were 17 months and 9 months, respectively, which were poorer than that of wild‐type group (*p* = 0.022 for BRAF‐mut; *p* < 0.0001 for NRAS‐mut). The median DFS of KIT‐mutant patients was 33 months, which was similar for wild‐type patients (*p* = 0.200). (Figure 2A) By contrast, no survival difference was found among wild‐type patients and those with different gene mutations in the IFN/OBS group. (Figure 2B).

**FIGURE 3:**
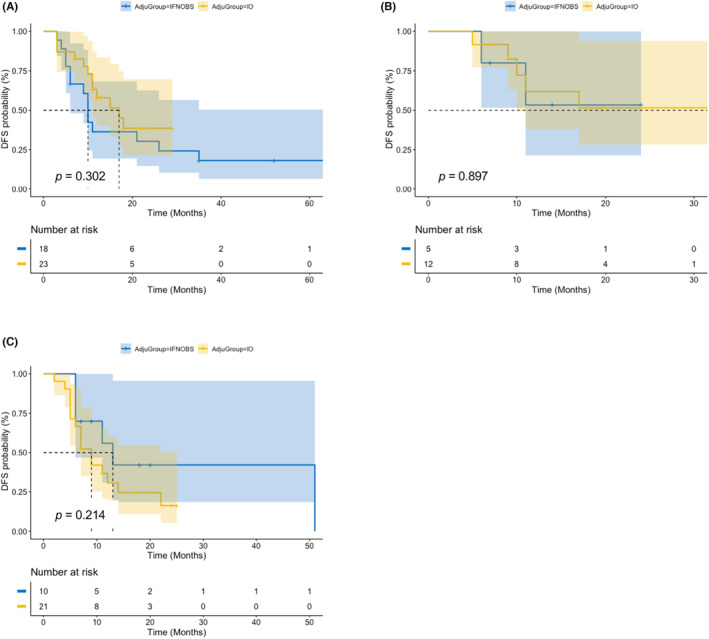
Kaplan–Meier plot curves for the disease‐free survival (DFS) of patients harbor different gene mutations (A: patients with BRAF mutations; B: patients with C‐KIT mutations; C: patients with NRAS mutations) in anti‐PD‐1 immunotherapy group and IFN/OBS group.

To further evaluate the efficacy of adjuvant immunotherapy with different genomic alternation, we compared the outcome of different treatments, respectively, in each gene mutation group. For patients with BRAF mutations, no statistically difference was observed although a trend of benefit showed in IO group in IFN/OBS group (median DFS: 17 vs. 10 months; *p* = 0.302, Figure 3A). Negative finding of DFS was shown in KIT‐mut patients either. (*p* = 0.897, Figure 3B). Interestingly for NRAS‐mut patients, median DFS in IO group was even poorer than in IFN/OBS group (9 vs. 13 months), although there was no statistical difference (*p* = 0.214, Figure 3C).

## 4 DISCUSSION

Adjuvant anti‐PD‐1 monotherapy identified no disease‐free survival benefit in BRAF mutant melanoma than IFN/OBS in our study. Due to the late launch to market of anti‐PD‐1 in China, although not routinely used, a small part of (*n* = 22) patients do have choose high‐dose interferon (HD‐IFN) as adjuvant therapy strategy after 2017. It is not appropriate to ignore these patients in a real‐world study. As we know, IFN has been conventionally utilized for decades, while yielding limited clinical benefits than observation.^26^, ^27^, ^28^ Hence, we combined the IFN group and observation group as one, regarding this group as traditional treatment group, to compare with anti‐PD‐1 group. Limited studies have specially investigated the association between the effectiveness of anti‐PD1 blockade and BRAF mutation status. Several randomized controlled clinical trials (RCTs) such as KEYNOTE‐054, CheckMate‐238, and IMMUNED have revealed that BRAF mutation status may not influence the efficacy of anti‐PD‐1 treatment in their subgroup analysis.^4^, ^5^, ^6^, ^29^ However, contradictory voices cannot be ignored. The CheckMate‐037 trial, which evaluated nivolumab versus chemotherapy in patients with advanced melanoma who progressed after anti‐CTLA‐4 treatment, noted statistically better ORR in BRAF‐wildtype group with nivolumab, but no statistical difference in the BRAF^V600^ mutant patients.^30^ Another retrospective study suggested no statistical difference in PFS or OS with ipi/nivo versus anti‐PD‐1 monotherapy in the BRAF^V600^ mutant advanced melanoma patients.^31^ Several preclinical studies may explain this phenomenon. The oncogenic signaling related to the BRAF^V600^ mutation can drive the transcription of multiple genes that promote immune suppression, which lead to a counteractive antitumor effect with immunotherapy.^32^, ^33^, ^34^ BRAF mutant tumors also tend to have a lower average tumor mutational burden (TMB) than BRAF wildtype patients.^35^ As we know, low TMB is considered as a contributing factor to low efficacy for immunotherapy.^36^ Even though BRAF mutate patients tended to have a longer DFS receiving immunotherapy than IFN or observation, no statistical difference was identified in this study. Fortunately, BRAF/MEK inhibitors combined adjuvant therapy is also an option for patients with BRAF^V600^ mutations.

The impact of NRAS mutations in anti‐PD‐1 adjuvant therapy of melanoma is highly debated. In a retrospective study which included 60 NRAS mutation and 169 NRAS‐wildtype melanoma patients, the NRAS mutation cohort showed a particularly marked benefit from anti‐PD‐1/PD‐L1 than wild type (clinical benefit rate, 73% vs. 35%). The authors contributed this result to the higher PD‐L1 expression rate in NRAS‐mutant melanoma.^12^ Another study reported a comparable response rate between two groups (21% vs. 13%, *p* = 0.210), while the median OS of NRAS‐mutant patients was significantly lower with 21 months compared to 33 months in NRAS‐wildtype patients (*p* = 0.034).^13^ In the POLARIS‐01 trial, which was conducted in Asian advanced melanoma patients, the ORR of NRAS‐mutant cohort receiving toripalimab was only 6.1%, indicating NARS mutation a potential resistance mechanism for immunotherapy.^37^ The current study also implied that NRAS‐mutant patients do not benefit from anti‐PD‐1 antibodies. One reason may be the lower tumor‐infiltrating lymphocyte (TIL) grade for NRAS‐mutant melanoma, which lead to a more immunosuppressed microenvironment that may affect its response to immunotherapies.^8^ Previous strategies have focused on posttranslational modification of NRAS (farnesyltransferase inhibitors). However, the experience in clinical trials were generally disappointed.^38^ Mutations in NRAS constitutively activate the MAPK signaling cascade by switching signaling from BRAF to CRAF, which in turn activates MEK.^39^, ^40^ Hence, blocking the downstream signaling partner, using MEK inhibitors is an attractive therapeutic strategy. A partial response rate of 20% and 3.7 months of PFS in NRAS‐mutant melanoma were reported in a phase II study using MEK inhibitor binimetinib.^41^ In the subsequent open‐label phase III clinical trial comparing binimetinib versus dacarbazine, binimetinib monotherapy resulted in 15% ORR and 2.8 months median PFS in NRAS‐mutant patients.^42^ The combination of MEK inhibitor and anti‐PD‐1 immunotherapy was considered as a promising option. However, the phase III clinical trial of atezolizumab plus cobimetinib in advanced melanoma failed to demonstrate superior survival over anti‐PD‐1 monotherapy.^43^ Other combination strategies, such as CDK4/6 inhibitors, autophagy inhibitors, or HDAC inhibitors, are also under investigation to overcome the subsequent monotherapy resistance and to prolong the survival.^44^, ^45^, ^46^

Another interesting result in our study is that patients with KIT mutations also showed no survival benefit from anti‐PD‐1 adjuvant therapy than IFN/OBS. To the best of our knowledge, similar studies are still blank, which may attribute to the low incidence of KIT mutations in nonacral cutaneous melanomas. One of the most promising strategies in place of anti‐PD‐1 antibodies is KIT inhibitor. A variety of clinical trials have been conducted to explore the therapeutic value of c‐KIT inhibitors in KIT‐mutant melanomas.^47^, ^48^, ^49^, ^50^, ^51^ Given these clinical trial results demonstrating clinical benefit of KIT inhibitors, the National Comprehensive Cancer Network (NCCN) guidelines recommended KIT‐targeted therapy as one of the second‐line systemic therapies for metastatic or unresectable melanoma with active KIT mutations. In addition, in vivo studies have demonstrated enhanced immune responses, including increased T‐cell activation, natural killer cell clonal expansion, and enhanced tumor antigen presentation with KIT inhibitors.^52^, ^53^, ^54^, ^55^ Although several case reports have indicated the efficacy of combined KIT inhibitors with immunotherapy in KIT mutant advanced melanoma, related RCTs are urgently required.^18^, ^56^, ^57^

Although our study sheds new light on the influence of gene mutations on adjuvant immunotherapy in melanoma, several limitations still exist. First of all, on account of the retrospective data source, the existence of an inevitable bias at present may warrant further prospective data afterward. Besides, although this study included a substantial number of patients, various subgroup analyses leaded to dispersion of patient numbers, which may also cause bias.

## 5 CONCLUSION

In this real‐world study, melanoma patients with BRAF, KIT, or NRAS mutations treated with anti‐PD‐1 adjuvant monotherapy had no statistical difference of DFS compared to patients received IFN or observation. Although no statistical difference was concluded, NRAS‐mutant patients treated with anti‐PD‐1 antibodies even showed a trend toward poorer survival compared to IFN treatment or observation. New treatment strategies are required to improve the outcomes of gene mutant melanoma.

## AUTHOR CONTRIBUTIONS

**Wei Sun:** Data curation (lead); funding acquisition (lead); writing – original draft (lead). **Yu Xu:** Project administration (equal); resources (equal); writing – review and editing (equal). **Wangjun Yan:** Data curation (equal); resources (equal). **Chunmeng Wang:** Data curation (equal); resources (equal). **Tu Hu:** Data curation (equal); resources (equal); software (equal). **ZhiGuo Luo:** Data curation (equal); resources (equal). **Xiaowei Zhang:** Data curation (equal); resources (equal). **Xin Liu:** Data curation (equal); resources (equal). **Yong Chen:** Funding acquisition (equal); investigation (lead); project administration (lead); supervision (lead); writing – review and editing (lead).

## FUNDING INFORMATION

This work was financially supported by the National Natural Science Foundation of China (Grant No. 82272857), the Lingang Laboratory (Grant No. LG‐QS‐202205‐11) andthe Shanghai Committee of Science and Technology, China (Grant No. 19411951700).

## CONFLICT OF INTEREST STATEMENT

The authors declare that they have no competing interests.

## ETHICS STATEMENT

This study was approved by the Ethics Committee of FUSCC. Each participant signed an informed consent document during the preoperative conversation.

## Supporting information


Table S1

## Data Availability

The corresponding authors are pleased to provide the original data to those who are interested.
