# Supplementary material for: A real‐world study of adjuvant anti‐PD ‐1 immunotherapy on stage III melanoma with BRAF, NRAS, and KIT mutations
Source: Cancer Med. 2023 Jul 5;12(15):15945–54. doi: 10.1002/cam4.6234 (PMC10469738; doi:10.1002/cam4.6234)
Supplement: Supplementary file 1 — Table S1 [file CAM4-12-15945-s001.docx]

| Gene | Exon | | n (%) | SNV | n (%) | Change | n (%) |
| --- | --- | --- | --- | --- | --- | --- | --- |
| BRAF | 15 | 41 (100%) | | V600 | 41 (100%) | V600E | 41 (100%) |
| NRAS | 2 | 9 (29.0%) | | G12 | 9 (29.0%) | G12D | 3 (9.7%) |
|  |  |  | |  |  | G12A | 2 (6.5%) |
|  |  |  | |  |  | G12C | 2 (6.5%) |
|  |  |  | |  |  | G12S | 1 (3.2%) |
|  |  |  | |  |  | G12V | 1 (3.2%) |
|  | 3 | 21 (67.1%) | | Q61 | 21 (67.1%) | Q61R | 10 (32.3%) |
|  |  |  | |  |  | Q61K | 9 (29.0%) |
|  |  |  | |  |  | Q61H | 2 (6.5%) |
|  | Ampification | 1 (3.2%) | |  |  |  |  |
| KIT | 11 | 11 (64.8%) | | L576 | 8 (47.1%) | L576P | 8 (47.1%) |
|  |  |  | | D572 | 1 (5.9%) | D572Y | 1 (5.9%) |
|  |  |  | | V559 | 1 (5.9%) | V559A | 1 (5.9%) |
|  |  |  | | Y553 | 1 (5.9%) | Y553D | 1 (5.9%) |
|  | 13 | 3 (17.6%) | | K642 | 2 (11.8%) | K642E | 2 (11.8%) |
|  |  |  | | Y646 | 1 (5.9%) | Y646D | 1 (5.9%) |
|  | 17 | 3 (17.6%) | | N822 | 3 (17.6%) | N882I | 2 (11.8%) |
|  |  |  | |  |  | N882K | 1 (5.9%) |
